# Dendritic Nonlinearities Reduce Network Size Requirements and Mediate ON and OFF States of Persistent Activity in a PFC Microcircuit Model

**DOI:** 10.1371/journal.pcbi.1003764

**Published:** 2014-07-31

**Authors:** Athanasia Papoutsi, Kyriaki Sidiropoulou, Panayiota Poirazi

**Affiliations:** 1 Institute of Molecular Biology and Biotechnology (IMBB) – Foundation for Research and Technology-Hellas (FORTH), Heraklion, Crete, Greece; 2 Department of Biology, University of Crete, Heraklion, Crete, Greece; Imperial College London, United Kingdom

## Abstract

Technological advances have unraveled the existence of small clusters of co-active neurons in the neocortex. The functional implications of these microcircuits are in large part unexplored. Using a heavily constrained biophysical model of a L5 PFC microcircuit, we recently showed that these structures act as tunable modules of persistent activity, the cellular correlate of working memory. Here, we investigate the mechanisms that underlie persistent activity emergence (ON) and termination (OFF) and search for the minimum network size required for expressing these states within physiological regimes. We show that (a) NMDA-mediated dendritic spikes gate the induction of persistent firing in the microcircuit. (b) The minimum network size required for persistent activity induction is inversely proportional to the synaptic drive of each excitatory neuron. (c) Relaxation of connectivity and synaptic delay constraints eliminates the gating effect of NMDA spikes, albeit at a cost of much larger networks. (d) Persistent activity termination by increased inhibition depends on the strength of the synaptic input and is negatively modulated by dADP. (e) Slow synaptic mechanisms and network activity contain predictive information regarding the ability of a given stimulus to turn ON and/or OFF persistent firing in the microcircuit model. Overall, this study zooms out from dendrites to cell assemblies and suggests a tight interaction between dendritic non-linearities and network properties (size/connectivity) that may facilitate the short-memory function of the PFC.

## Author Summary

Working memory, the ability to retain information for a short period of time, is a fundamental cognitive function that shapes behavior. The cellular correlate of working memory is the prolonged spiking (persistent) activity of neurons in the prefrontal cortex. Impairments of prefrontal cortex functionalities and working memory have been associated with a variety of cognitive disorders, such as schizophrenia, the attention deficit hyperactivity disorder, and drug addiction. Hence, understanding how neurons embedded in the local circuitry support and maintain persistent activity is of outmost importance. Our work uses a multi-level integrative approach spanning from the dendritic, to the neuronal and network levels to identify the key biophysical and anatomical mechanisms contributing to persistent activity, leading to a number of high impact findings: it predicts a tradeoff between dendritic regenerative events and the size of a network expressing persistent activity. It also proposes when and how the persistent state can be stabilized, opening new avenues for pharmacological interventions. Finally, it describes decoding mechanisms for upcoming ON/OFF state transitions, furthering our understanding of information processing in the PFC and shedding new light on the emergence of anticipatory behaviors.

## Introduction

Small, tightly interconnected “clusters” of cortical neurons have recently been discovered in regions such as the visual, somatosensory and prefrontal cortex (PFC) [1]–[6], yet their role in cognitive processes remains unexplored. In the PFC, such microcircuits have been suggested to participate in persistent activity, the cellular correlate of working memory, but this hypothesis has not been rigorously tested [7], [8]. Towards this goal, Papoutsi and colleagues developed a layer 5 (L5) PFC microcircuit model, heavily constrained against experimental data, and showed that such microcircuits can serve as tunable modules of persistent activity [9]. What remain unclear are the biophysical and anatomical mechanisms that allow the induction (ON) and can cause termination (OFF) of persistent firing in such modules.

Previous studies have uncovered the NMDA synaptic current [10], [11] and the delayed afterdepolarization (dADP) [12] as two important biophysical mechanisms that contribute to persistent activity initiation. However, the structure and size of the network studied varied greatly, from a couple of cells [13] to networks of hundreds to thousands of neurons (*in silico*) [14], [15] along with networks of unknown size in the slice preparation or *in vivo*. Moreover, in the majority of these studies, pharmacological manipulations (e.g. carbahol addition [16]) and/or unrealistic connectivity properties (e.g. long conduction delays [13]) were needed for the phenomenon to emerge. As a result, the minimum size of a network capable of expressing persistent activity under physiological conditions remains unknown, yet critical for understanding the mechanisms underlying its induction [17].

Like network size, the role of dendritic nonlinearities in persistent activity emergence is also ambiguous. First, the NMDA receptors, which are primarily located in the dendrites of cortical pyramidal neurons, where found to be imperative for the *in vivo* initiation of persistent activity in the PFC [10]. Second, the generation of dendritic plateau potentials at the basal dendrites of L5 PFC neurons [18], [19] has been suggested to underlie the somatic depolarization observed during Up states [20]. However, while these studies concern a known player – the NMDA receptor - a link between dendritic regenerative events and persistent activity emergence has yet to be established.

Finally, little is known about how key characteristic features of persistent activity like stimulus-specificity, resistance to distracters and termination induced by behavioral actions [21] can be implemented by neural tissue. For example, the only candidate mechanism for termination is inhibitory input which was shown to cease Up states [22]. Regarding stimulus-specificity, Sidiropoulou and Poirazi, 2012 used a computational model of a single L5 PFC pyramidal neuron to show that location of activated synapses along the basal dendrites and action potential timing could serve as encoding and decoding mechanisms, respectively, of stimulus-selective induction [23]. Identifying such information in the response pattern of these neurons is particularly important as it may signal the upcoming state transitions to downstream neurons, setting the ground for the subsequent behaviour actions that will terminate persistent firing. However, whether such mechanisms are also relevant at the network level or whether other mechanisms are implicated in not known.

Here, we use our recently developed microcircuit model [9] to investigate the mechanisms that underlie persistent activity emergence (ON) and termination (OFF) at the dendritic, neuronal and network levels and search for the minimum network size required for expressing these states within physiological regimes. Moreover, we search for mechanisms that may underlie persistent activity maintenance upon presentation of distracting stimuli and identify network characteristics that code for the upcoming state transitions.

## Results

**Figure 1:**
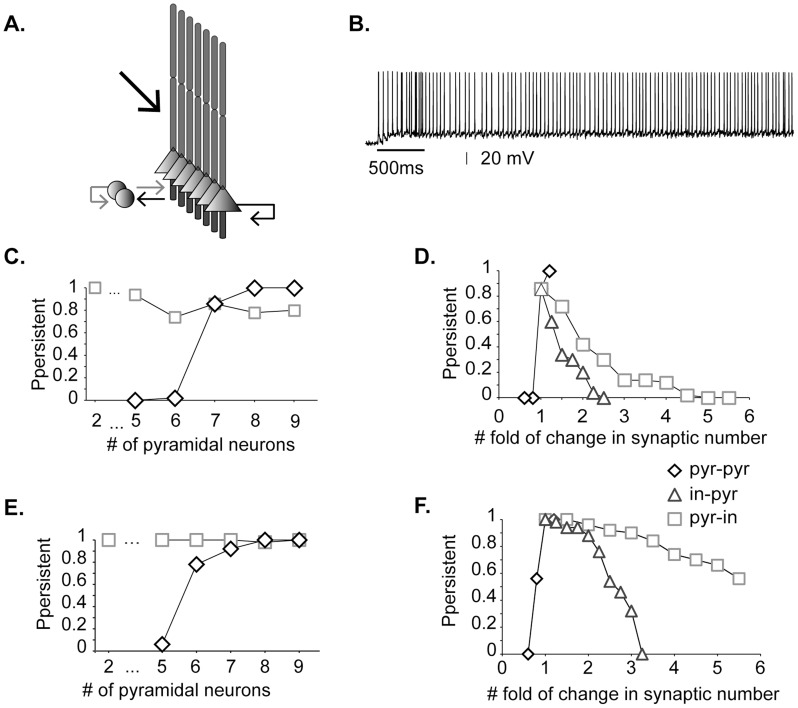
Synaptic drive underlies persistent activity induction. **A.** The microcircuit model includes seven pyramidal cells and two inhibitory interneurons reciprocally connected. Black arrows: excitatory connections, grey arrows: inhibitory connections (adapted with permission from [9]). Persistent activity is induced following external stimulation of the proximal apical dendrites (thick black arrow). **B.** Indicative trace of persistent activity in the model (iNMDA-to-iAMPA ratio = 2.3 and dADP = 0 mV). Bar shows stimulus presentation. **C.** Probability of persistent activity induction as a function of the number of pyramidal neurons in the microcircuit. Diamonds correspond to increasing numbers of cells whereby each pyramidal-pyramidal neuron pair is connected with 5 synapses. Squares correspond to increasing numbers of cells in which the total number of excitatory connections per neuron is fixed to 31 (corresponding to a network with 7 pyramidal neurons). **D.** Probability of persistent activity emergence as a function of changes in the number of synapses between pyramidal neurons (pyr-pyr, diamonds) and between interneurons and pyramidal neurons (in-pyr, triangles/pyr-in, squares). **E, F.** Same as in C, D, when in *vivo-like* background synaptic activity was activated.

Simulations were performed in a microcircuit model [9] composed of 7 pyramidal neurons and 2 inhibitory interneurons, all reciprocally connected (Figure 1A). Stimulus-induced persistent activity emerged (Figure 1B) when the NMDA current was enhanced by ∼75–100% (iNMDA-to-iAMPA = 1.9 or 2.3, control value = 1.1), simulating the increase of the NMDA current in L5 PFC pyramidal neurons due to dopamine release [24], [25], the recruitment of extrasynaptic receptors [19] or due to glia related-processes [20]. Induction resulted from synaptic stimulation (10 pulses at 20Hz, 90 excitatory synapses) at the proximal dendrites of each pyramidal model neuron. A total of 50 simulation trials were performed for each condition, whereby the location of incoming and connecting synapses varied randomly along the basal dendrites. Additional sources of noise included membrane fluctuations, conductance delay variability, etc. (see Materials and Methods) and were used to represent the dynamics of such networks. *In vivo-like* background activity was also implemented for certain experiments. The iGABA_B_-to-iGABA_A_ ratio was 0.2 and the dADP mechanism was deactivated (control case), unless reported otherwise.

### Network size vs. network connectivity

To investigate the effect of network size on persistent activity emergence, we varied the number of pyramidal neurons in the microcircuit and recorded the result of this manipulation on the probability of induction. We found that reducing the number of pyramidal neurons from 7 to 5 completely abolished persistent activity, whereas adding more neurons increased the probability of induction to 100% (Figure 1C, diamonds). The latter could be due to stronger synaptic drive within the network (a connectivity effect) or due to having more neurons that propagate signals (a size effect). To discriminate between these two possibilities, we varied the network size while keeping the number of recurrent connections per neuron fixed to that of a size 7 network (31 synapses per neuron: 6×5 pyramidal-to-pyramidal inputs plus 1 autapse). Persistent activity emerged in all cases tested (Figure 1C, squares), even in a microcircuit of size 2. On the contrary, changing the synaptic drive of each neuron (in a network of size 7) had a strong effect: reducing the pyramidal-to-pyramidal synaptic contacts by 20%, from 5 to 4 (total inputs per neuron: from 31 to 25) abolished persistent activity, whereas the respective increase in connections from 5 to 6 (total inputs per neuron: from 31 to 37) led to 100% probability of induction (Figure 1D, diamonds). Varying the connectivity strength between pyramidal and interneurons had less pronounced effects: persistent activity could emerge even when the pyramidal-to-interneuron connections tripled (Figure 1D, squares) or the interneuron-to-pyramidal connections doubled (Figure 1D, triangles).

The validity of these findings was also tested under conditions that more closely approximate the *in vivo* situation, where neurons constantly receive synaptic barrages that alter their dynamics. Background synaptic activity as reported *in vivo* during quiet wakefulness and not under anaesthesia [26], [27] was added to both pyramidal neurons and interneurons (Figure S1B) and the same experiments were repeated. Results were very similar to the previous analysis. Excitatory synaptic transmission was still the determinant factor for persistent activity emergence (Figures 1E, F). The main effects of background synaptic activity where: (a) to slightly reduce the synaptic drive required for persistent activity (emergence in a network with 6 pyramidal neurons instead of 7, Figure 1E) and (b) to increase the tolerance of persistent activity emergence to changes in inhibitory transmission (connection strength between pyramidals and interneurons, Figure 1F). Overall, these results suggest that the strength of excitatory-to-excitatory transmission, as opposed to the network size, is the crucial factor for persistent activity induction in the microcircuit, under both *in-vitro* and *in-vivo* like conditions.

### Network size vs. NMDA spikes

Given that excitatory-to-excitatory transmission is crucial for persistent activity induction and NMDA receptors play a key role in shaping excitatory synaptic transmission in L5 PFC pyramidal neurons [19], [28], we next examined their contribution to the size vs. synaptic drive argument. Activation of NMDA receptors was recently found to be imperative for persistent activity emergence *in vivo* [10] and the generation of Up-states in acute slices [20], [29], [30]. Since both of these phenomena are characterized by long-lasting depolarizations, it can be assumed that the role of NMDA currents is to provide or sustain these depolarizations through regenerative dendritic events such as NMDA spikes [19]. We thus investigated whether and how the generation of NMDA spikes may influence persistent activity induction in the microcircuit model.

We first assessed whether NMDA spikes are inducible in our pyramidal neuron models using four different iNMDA-to-iAMPA ratios: 1.1, 1.5, 1.9, and 2.3. In all cases, only the NMDA current increased and the ratios were calculated under voltage clamp conditions (Figure S1A). Stimulation of increasing number of synapses (5–50, with step of 5) at the basal dendrite of a single pyramidal neuron with 2 pulses at 50Hz led to a non-linear increase in the somatic EPSP amplitude for ratios 1.9 and 2.3 (Figure S2A), that was also evident in the EPSP half width (Figure S2B). Moreover, comparison of model and experimental data regarding the EPSP amplitude and half width (for a ratio of 2.3) measured under single-pulse and paired pulse (at 50Hz) stimulation revealed a close mapping between simulated and experimental attributes of synaptic integration in basal dendrites of L5 PFC pyramidal neurons [19] (Figure S2E). These findings are characteristic of dendritic NMDA spike generation.

**Figure 2:**
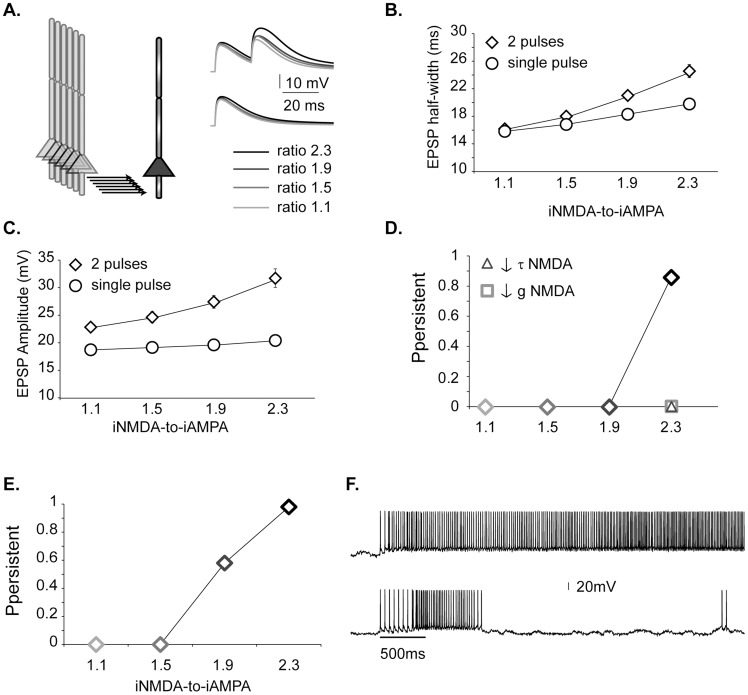
iNMDA-to-iAMPA ratio controls persistent activity induction. **A.** Indicative basal EPSP traces from a single pyramidal neuron for different iNMDA-to-iAMPA ratios (1.1, 1.5, 1.9 and 2.3) after stimulation of 31 synapses with 2 pulses at 50 Hz (upper panel) or 1 pulse (lower panel), under conditions of Na^+^ channel blockade. **B.** Mean ± std of the dendritic EPSP half width. The EPSP half width increases non-linearly for ratios above 1.5, only for the paired pulse protocol. **C.** Mean ± std of the dendritic EPSP amplitude for the same conditions as in B. Note the increase in amplitude only for the paired pulse protocol. **D.** Probability of persistent activity emergence for different iNMDA-to-iAMPA ratios, calculated over 50 trials. Triangle: reduced NMDA decay time constant (from τ = 107 ms to τ = 18 ms). Square: blocked NMDA receptors (90% reduction in conductance, while compensating for reduced excitability by increasing the AMPA conductance). **E.** Probability of persistent activity emergence for different iNMDA-to-iAMPA ratios calculated over 50 trials, in the presence of background synaptic activity. **F.** Indicative voltage traces from a persistent trial (top) and a transient stimulus response (bottom) in the presence of background synaptic activity (iNMDA-to-iAMPA ratio = 1.9). Bar indicates stimulus presentation.

In addition, the ability to induce NMDA spikes in a given pyramidal neuron with input from connecting synapses alone (6 neurons×5 inputs +1 autapse = 31 synapses) was assessed. Representative traces resulting from the activation of 31 synapses (2 pulses at 50Hz: diamonds or 1 pulse: squares) in the basal dendrite of a pyramidal neuron model, under blockade of Na^+^ channels are shown in Figure 2A. The dendritic EPSP half width and amplitude are shown in Figures 2B and 2C, respectively for both the paired (at 50Hz) and single pulse protocols. Enhancement of dendritic EPSP amplitude and half width, reminiscent of NMDA spike generation [19], was seen primarily for a ratio of 2.3 (small increases are also seen for a ratio of 1.9) under the paired -but not the single- pulse stimulation, suggesting the occurrence of NMDA spikes. These results are in very good agreement with experimental recordings [19] (Figure S2).

Emergence of persistent activity was strongly correlated with the generation of NMDA spikes. As shown in Figure 2D, lack of prominent NMDA spikes (ratio 1.1–1.9) was associated with zero probability of persistent activity, whereas generation of large NMDA spikes (ratio 2.3) was associated with an induction probability of 86%. Importantly, reducing the NMDA decay time constant (from τ = 107 ms to τ = 18 ms, Figure 2D, triangle) or blocking the NMDA receptors (90% reduction in conductance, while compensating for reduced excitability by increasing the AMPA conductance) under conditions that normally supported persistent activity (Figure 2D, square) also abolished the persistent state.

### Ongoing network activity facilitates NMDA spike generation

The above experiments were repeated in the presence of background synaptic activity to establish their validity under *in-vivo like* conditions. The only difference observed was a reduction in the amount of NMDA current required for persistent activity emergence: the induction probability for ratio of 1.9 climbed from zero to 0.58 (Figure 2E). These findings suggest that background synaptic input facilitates persistent activity induction by enhancing NMDA spikes appearing at a ratio of 1.9 (Figure 2B, Figure S2B), which would otherwise be ineffective. Representative traces with and without persistent activity in the presence of background synaptic input for the 1.9 ratio are shown in Figure 2F.

**Figure 3:**
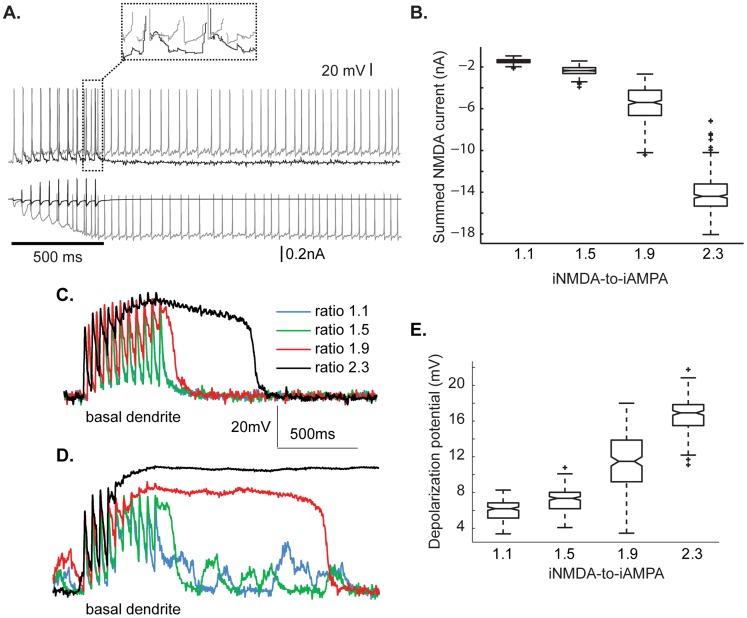
NMDA spikes underlie persistent activity induction. **A.** Top panel: Indicative traces of transient stimulus response for a ratio of 1.1 (black) and of persistent activity corresponding to iNMDA-to-iAMPA ratio of 2.3 (grey). Bottom panel: net NMDA current the neuron receives for the ratio 1.1 (black) and 2.3 (grey). Bar indicates stimulus presentation. **B.** Box plot showing the cumulative sum of the net iNMDA during the 500 ms of stimulus presentation, for the different iNMDA-to-iAMPA ratios. **C–D.** Depolarization recorded at the basal dendrite in the absence (C) and in the presence (D) of background synaptic activity of the ‘silent’ pyramidal neuron embedded in the microcircuit, for the different ratios used. **E.** Box plot of the depolarizing potential during the last 100 ms of the stimulus (dotted box in A). Note the generation of a large plateau potential (∼16 mV) for the 2.3 ratio.

To investigate whether NMDA spikes are generated during persistent activity, we evaluated whether individual pyramidal neurons express such NMDA spikes when embedded in the microcircuit model and not in isolation, as was done above. Persistent activity induction in the microcircuit was associated with a larger depolarization at the soma (Figure 3A, top and Figure 3E) and a larger inward NMDA current (Figure 3A, bottom and Figure 3B), compared to the non-persistent state. Representative traces for the two cases are shown in Figure 3A (black trace: ratio 1.1, grey trace: ratio 2.3). To investigate whether this synaptic current supports NMDA spikes at the basal dendrites, we added to the microcircuit a pyramidal neuron in which we blocked the somatic and axonal fast sodium channels. This ‘silent’ pyramidal neuron received the same synaptic activity as the other pyramidals, but did not contribute to the microcircuit activity. As shown in Figure 3C, the characteristic depolarization plateau potential of NMDA spikes is absent for ratios 1.1 and 1.5, emerges with small width for a ratio of 1.9 and becomes pronounced for a ratio of 2.3. In the presence of background synaptic activity, these features appear at smaller ratios: small plateau potentials are evident at a ratio of 1.5, they become pronounced at a ratio of 1.9 and they always lead to persistent activity for a ratio of 2.3 (Figure 3D). These results illustrate that NMDA spikes do emerge in pyramidal neurons participating in the microcircuit, under conditions that enable persistent activity induction (ratio of 1.9 & 2.3).

### NMDA spikes underlie persistent activity via the build-up of a somatic plateau potential

Finally, we tested whether the mechanism of action of NMDA spikes is to provide long-lasting somatic depolarizations on top of which persistent activity can ride. Specifically, we measured the somatic depolarization of a pyramidal neuron model during the last 100 ms of stimulus presentation (Figure 3A, dotted box) for the four different ratios. Generation of NMDA spikes for a ratio of 2.3 (calculated over 50 trials) induced, on average, a large plateau potential (∼16 mV) at the soma; smaller plateaus were seen for ratios of 1.1–1.9 (Figure 3E). This depolarized state, which is not seen for small ratios, is proposed to underlie the persistent spiking activity. To further investigate this hypothesis we asked whether the 100 ms depolarizing potential is also different between trials that led to persistent firing vs. trials that didn't, this time for a fixed iNMDA-to-iAMPA ratio. Indeed, for a ratio of 2.3, this plateau potential was significantly larger in the persistent compared to the transient response trials (p value<0.001) (Figure S4A).

These results suggest that somatic depolarizations resulting from NMDA spike generation may underlie persistent activity. If this was truly the case, injection of a current at the soma could potentially substitute the need for NMDA spikes at the basal dendrites. To test this hypothesis, we blocked NMDA receptors in all pyramidal neurons and delivered a depolarizing current throughout the stimulus and delay periods. We found that, currents resulting in somatic depolarization potentials similar to the ones seen for a ratio of 2.3 (16mV) also supported persistent activity (Table 1). This raised the issue that other intrinsic mechanisms may support persistent activity in small microcircuits, if they can build a similarly-sized depolarizing plateau potential at the soma. To address this question we blocked NMDA receptors in all pyramidal neuron models and independently enhanced the conductance of each excitatory ionic mechanism by a factor of 2–5. Examined mechanisms included the Na_f_, Na_P_, Ca_L_, Ca_T_, Ca_R_, Ca_N_, as well as the conductance of the h current that has been shown to participate in persistent activity induction [31]. None of these manipulations resulted in persistent activity, reinforcing our previous findings that NMDA spikes at the basal dendrites support persistent spiking activity in small microcircuits, via the build-up of long-lasting depolarizing plateau potentials [32].

**Table 1:** Depolarizing current supports persistent activity in the same range as NMDA spikes.

Amplitude (nA)	0.1	0.15	0.2
Depolarization (mV)	8	12	16
Persistent	0	0	1

The only mechanism able to replace NMDA spikes was the dADP, a mechanism activated by cholinergic input in L5 PFC pyramidal neurons, which was previously linked to persistent activity by us and others [12], [23]. However, it should be noted that the amplitude of the dADP required for persistent activity emergence under NMDA blockade was 15 mV, namely much larger than the physiologically reported values (1–4 mV [23]). In agreement with prior work [23], these results suggest that while intrinsic ionic conductances, and particularly the dADP, can contribute to persistent activity, NMDA receptors are crucial for its emergence.

In sum, our simulations predict that dendritic nonlinearities alone, through the generation of NMDA spikes and a subsequent build up of somatic depolarization, act as a switch for entering a sustained firing state in L5 PFC microcircuits.

### NMDA is critical for small but not large scale networks

**Figure 4:**
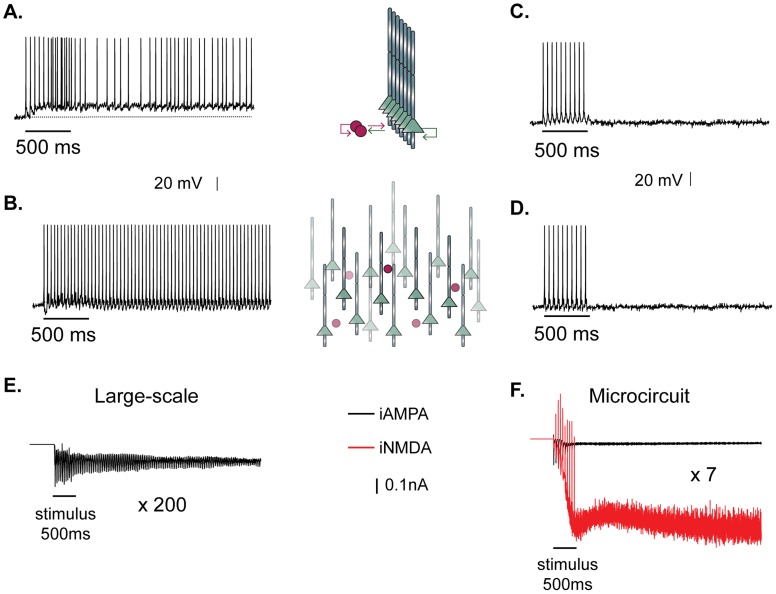
Depolarizing plateau underlies persistent activity only in the presence of NMDA receptors. **A.** Persistent activity after stimulation at the proximal dendrites of the microcircuit (iNMDA-to-iAMPA ratio = 2.3, latency of excitatory synaptic transmission: 1.7±0.9 ms). Bar indicates stimulus presentation. Note the generation of a depolarizing somatic plateau potential. **B.** Persistent activity after stimulation at the proximal dendrites of a large scale network (number of pyramidal neurons: 200, number of interneurons: 50) while blocking NMDA receptors and increasing the latency of excitatory synaptic transmission to 40±10 ms. Note the absence of the depolarizing plateau potential. **C.** Persistent activity fails to emerge in the microcircuit when NMDA receptors are blocked and the latency of excitatory synaptic transmission is increased to 40±10 ms, as in **B.** **D.** Persistent activity fails to emerge in the large scale network when NMDA receptors are blocked and the latency of excitatory synaptic transmission is reduced to 1.7±0.9 ms. Bar indicates stimulus presentation. **E.** Average net AMPA current a pyramidal neuron receives from all other pyramidal neurons in the large scale network in the trials that led to persistent activity. **F.** Average net AMPA current (black trace) and the net NMDA current (red trace) a pyramidal neuron receives from all other pyramidal neurons in the microcircuit in the trials that led to persistent activity.

The predicted dependence on NMDA dendritic spikes may seem contradictory to previous work, whereby both large- [15] and small- scale neuronal network models [13] without NMDA receptors supported persistent activity. However, those models were not biophysically constrained in several aspects, including their connectivity properties. Moreover, blockade of NMDA receptors in the PFC was shown to abolish prolonged spiking activity [10], [33], suggesting that this dependence may be a region-specific effect. To further investigate this issue, we simulated a large scale network of fully connected 250 neurons (200 pyramidal and 50 interneurons) whereby NMDA and GABA_B_ receptors were completely blocked (as in earlier reports) and constraints regarding synaptic delays between pyramidal neurons were relaxed (delays for excitatory-to-excitatory connections were drawn from a Gaussian distribution with μ = 40 ms and σ = 10 ms). We found that the resulting asynchronicity in conjunction with the much larger size of the network were sufficient for persistent activity to emerge (Figure 4B) with high probability (82%), in agreement with earlier work [13]. Note the elimination of the somatic plateau potential during persistent firing generated in our large-scale networks (Figure 4B) compared to the microcircuit (Figure 4A). To test whether asynchronicity produced by long conduction delays was sufficient to replace NMDA-induced depolarizations, we blocked NMDA receptors in the microcircuit model and allowed for conduction delays similar to the ones used in Figure 4B. In this case, persistent activity could not be induced in any of the trials tested (Figure 4C). Similarly, we asked if reverberating activity in a large scale network with short conduction delays (similar to the ones used in the microcircuit) could support persistent firing under NMDA blockade (Figure 4D) and again failed to see sustained responses.

To elucidate the mechanisms that underlie persistent firing in the two network configurations, we recorded the net excitatory synaptic current to each pyramidal neuron, under conditions leading to persistent activity emergence with the same probability (86% for the microcircuit and 82% for the large-scale). As shown in Figure 4E–F, the net excitatory synaptic current per neuron is considerably larger in the microcircuit compared to the large scale network and is in great part mediated by the NMDA receptors (Figure 4F, red trace). Note that the total synaptic current that flows through *all* pyramidal neurons is, of course, much greater in the large scale network than in the microcircuit. These findings suggest that in small, biophysically constrained PFC microcircuits, where conduction delays are short, synaptic input to pyramidal neurons through NMDA receptors is necessary for persistent activity induction. This necessity disappears in large scale networks whereby long conduction delays in conjunction with multiple reverberating connections are sufficient to bridge depolarizations over time, thus prolonging spiking.

### Persistent activity termination is strength and dADP dependent

**Figure 5:**
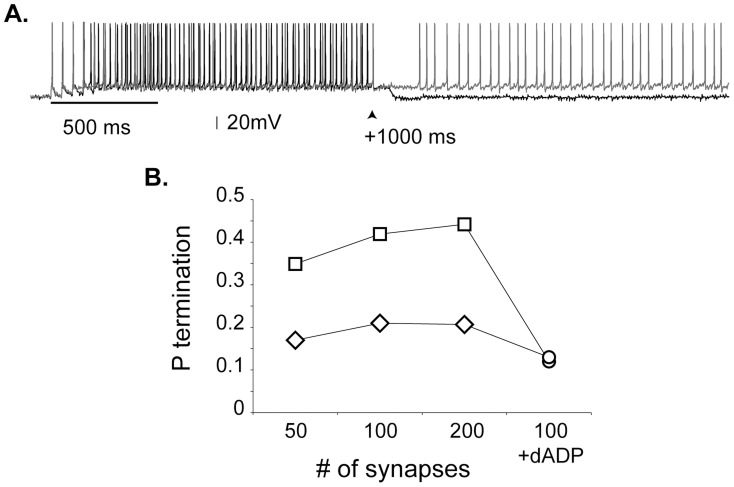
Stability of persistent activity depends on the background synaptic activity and the activation of the dADP current. **A.** Indicative traces of persistent activity termination (black) and maintenance (grey) when a second stimulus (100 Hz for 100 ms) arrives 1 sec (arrow) after the inducing stimulus (dADP = 0 mV, iNMDA-to-iAMPA: 2.3). **B.** Probability of persistent activity termination as a function of the number of synapses delivered to the interneuron (50-100-200) under control condition (iNMDA-to-iAMPA: 2.3, dADP = 0 mV, squares) and in the presence of background synaptic input (iNMDA-to-iAMPA: 1.9, dADP = 0 mV, rhombus). dADP activation (absence of background activity: iNMDA-to-iAMPA: 1.9, dADP = 2 mV, presence of background activity: iNMDA-to-iAMPA: 1.5, dADP = 2 mV) is shown with circles. The different iNMDA-to-iAMPA conditions were used to ensure similar induction probabilities.

The persistent activity recorded during working memory terminates normally upon the execution of motor actions [34] or prematurely as a result of distracting stimuli, in which case performance drops significantly [35]. However, the mechanisms underlying persistent activity termination remain unclear. Inhibition is currently the primary candidate, as it has been found that Up states, a condition similar to persistent firing, are terminated by activation of interneurons [22]. Since PFC interneurons receive feed forward excitation during working memory tasks [36], we investigated their role in persistent activity termination. Delivery of a second excitatory stimulus (10 events at 100Hz) to the interneuron models one second after induction resulted in termination of persistent with a probability less than 0.5. Representative traces of a terminating and a stable trial are shown in Figure 5A. Increased inhibitory input resulted in a slight increase of the termination probability whereas activation of the dADP mechanism had the opposite effect (Figure 5B). Specifically, dADP activation (2 mV) led to a significant decrease (∼21%) in the termination probability (Figure 5B, circles). These results are the first to propose a role of the dADP mechanism in the stabilization of persistent activity. Since the dADP primarily emerges following acetylcholine or glutamate action and is modulated by dopamine [12], our data suggest that neuromodulatory effects are likely to have a key role in the maintenance of persistent firing. Finally, termination was significantly harder in the presence of ongoing network activity (Figure 5B, diamonds), suggesting a new role for this activity in stabilizing persistent firing. In sum, these results show that termination of persistent activity depends on the strength of the synaptic input and is negatively modulated by dADP activation.

### Predicting state transitions

The abovementioned experiments showed that in small, biophysically constrained PFC microcircuits, dendritic events underlined by NMDA spikes build a somatic depolarization plateau on top of which stable persistent activity rides. Termination of this activity can be achieved via an inhibitory input. To substantiate our results regarding the key role of synaptic currents in persistent firing, we asked whether the response properties of the microcircuit (i.e. synaptic mechanisms and activity features) during stimulus presentation contained predictive information regarding both the induction and the termination of the persistent state. Termination was caused, as previously, by a second stimulus delivered to the interneurons 1 s after the inducing stimulus (100 synapses activated with 10 events at 100Hz). Induction and termination were evaluated under control conditions over 500 and 423 trials, respectively.

We used a linear SVM classifier (see Methods for details) to identify features of the stimulus-induced response that can serve as predictive markers for the microcircuit output. The examined features included measures of a) network spiking activity, b) single-cell spiking activity and c) single-cell synaptic currents. For each feature tested, the SVM was trained with 100 trials (training set) exhibiting the desired phenotypes (i.e. persistent activity vs. transient response or terminated vs. stable persistent activity) and prediction accuracy, sensitivity and specificity were estimated on a set of 30 previously unseen trials (test set). In all cases, a strict threshold of 70% sensitivity and specificity was used for the identification of informative features.

**Figure 6:**
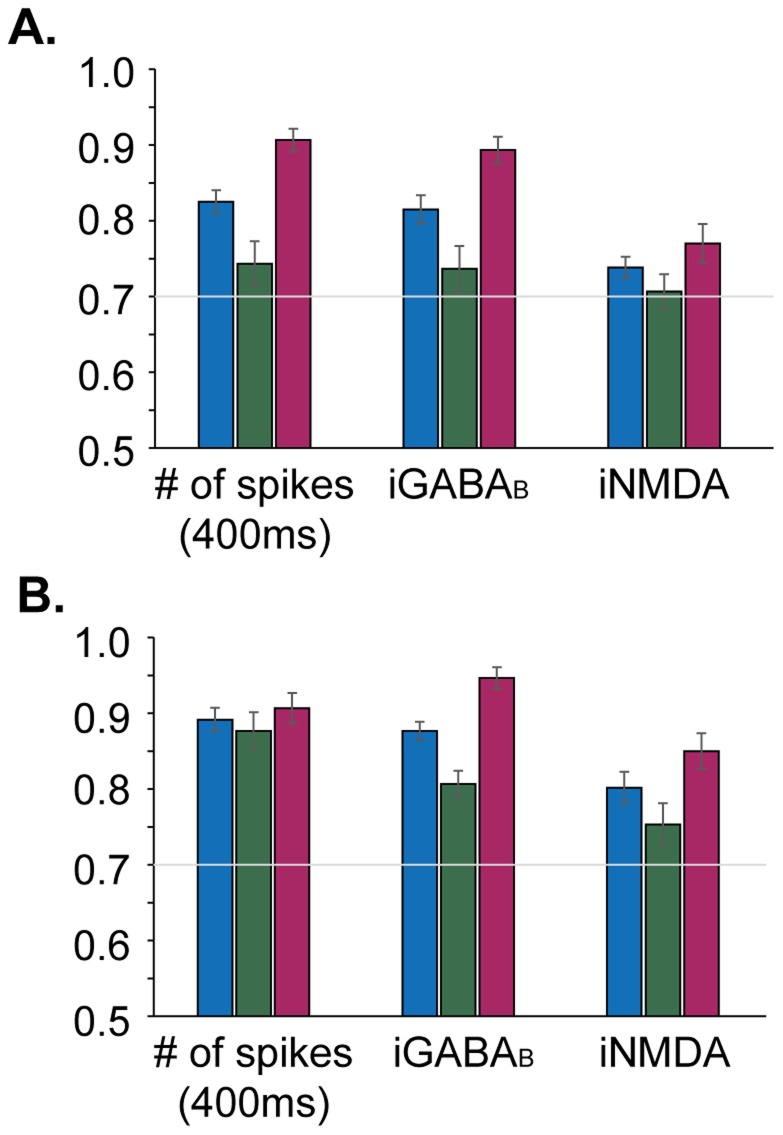
Predicting state transitions. Certain features of the microcircuit measured during the presentation of the inducing stimulus carry predictive information regarding an upcoming switch to/from the persistent activity state. Blue bars: mean accuracy ± standard error, green bars: mean sensitivity ± standard error, red bars: mean specificity ± standard error. **A.** Predictive features for the emergence of persistent activity. **B.** Predictive features for the termination of persistent activity. **A**, **B.** The total spiking activity during the first 400 ms of the stimulus presentation and the filtered iGABA_B_ and iNMDA at a single pyramidal neuron during the 500 ms of the stimulus predict induction and termination of persistent activity.

We started by examining the predictive power of synaptic currents measured at a single pyramidal neuron. We found that both NMDA-mediated slow excitation and GABA_B_-mediated slow inhibition coded for the upcoming state transitions. Specifically, the iGABA_B_ during stimulus presentation predicted induction and termination with an accuracy of 82%±8% (Figure 6A) and 88%±5% (Figure 6B), respectively. Although counterintuitive, the total GABA_B_ current was significantly larger in persistent than transient response trials (p value<0.001, non-parametric parametric U test, Figure S3C). The same was true for stable compared to terminated trials (p value<0.001, non-parametric parametric U test, Figure S3D).

A similar trend was seen for the NMDA current, which predicted induction and termination with accuracies of 74%±6% (Figure 6A) and 80%±9% (Figure 6B), respectively. In this case, the total NMDA current during stimulus presentation was significantly larger in persistent than in transient response trials (p value<0.05, non-parametric parametric U test, Figure S3E) but not in stable vs. terminated trials (p value>0.05, non-parametric parametric U test, Figure S3F). Note that, in order to study the impact of their slow kinetics, the NMDA and GABA_B_ currents were filtered using a Butterworth low pass filter. Indicative traces before and after the filtering of the NMDA and GABA_B_ currents are shown in Figure S3A, B.

The predictive power of NMDA and GABA_B_ currents during stimulus presentation does not explain *how* these mechanisms determine the induction and termination of persistent activity. We hypothesize that their interactions shape the somatic plateau potential towards the end of the stimulus as described in Figure 3E. We thus tested whether this plateau potential is significantly different not only between persistent and transient response trials as shown in Figure S4A, but also between stable and terminated trials. Indeed, the somatic depolarization (measured in the last 100 ms of the stimulus presentation) in stable trials was significantly larger than in terminated ones (p value<0.001) (Figure S4B), suggesting that the magnitude of the somatic membrane depolarization is a determining factor for both the emergence and termination of persistent firing in the PFC microcircuit.

Finally, previous work from our lab showed that temporal features (first spike latency and first inter-spike-intervals) of the stimulus-induced response of a single L5 PFC pyramidal neuron model code for an upcoming transition to a persistent state [23]. We thus tested whether this type of coding is preserved at the microcircuit level. State transitions in the microcircuit could not be predicted by the first few ISIs of the pyramidal neuron responses. A possible explanation is the lack of a detailed dendritic morphology for pyramidal neurons, which could account for different responses generated by location specific inputs, as previously argued [23]. However, we found that both the induction and the termination of persistent activity could be accurately predicted by the total number of spikes from all pyramidal neurons measured over the first 400 ms of the stimulus presentation. This feature predicted persistent activity emergence with an accuracy of 83%±7% (Figure 6A) and termination with an accuracy of 89%±6% (Figure 6B), respectively. In both cases, the total number of pyramidal neuron spikes during stimulus presentation was higher for the persistent vs. transient (p value<0.001, Figure S4C) and stable vs. terminated (Figure S4D) states, respectively.

Overall, our findings show that both spiking characteristic of the network activity and slow synaptic currents through their effect on somatic membrane depolarization, mediate induction as well as termination of persistent activity in the microcircuit model.

## Discussion

In this study we used a recently developed biophysical model of a L5 PFC microcircuit [9] to investigate the contributions of dendritic, somatic and network events to persistent activity emergence (ON) and termination (OFF).

### NMDA spikes vs. network properties

Dendrites of PFC pyramidal neurons have distinct NMDA receptor properties with enriched NR2B subunits and slower kinetics compared to sensory areas [28], [37]. In fact, the expression of mRNAs for NMDA subunits is higher in the PFC than in other regions of the human neocortex [38]. How exactly do NMDA receptors contribute to PFC function? Computational studies have traditionally considered NMDA current as a slow mechanism that provides stability to persistent activity in large scale networks [11]. This view has recently been challenged as oversimplified, involving asymmetric contributions of NMDA receptors in excitatory *versus* inhibitory pathways [39]. Regenerative, non-linear dendritic integration that depends on the iNMDA-to-iAMPA ratio [32] has been recorded *in vitro* in L5 PFC pyramidal neurons, as well as in many other areas [40]–[42]. In these neurons, enhancement of the NMDA conductance needed for the emergence of the non-linear behavior was shown to depend on the recruitment of extrasynaptic NMDA receptors [19], through glia-related events [20], or could be due to dopamine-dependent increase in the NMDA conductance [24].

Our study predicts that the regenerative events occurring in the basal dendrites of PFC pyramidal neurons, in particular NMDA spikes, gate the induction of persistent firing in a biophysically validated PFC microcircuit, but not in large scale neuronal networks with relaxation of biophysical constrains. A number of studies have suggested that NMDA receptors are critical for persistent firing [10], [11], [39], however, our study is the first to provide a direct link between NMDA spike generation in dendrites and persistent activity induction. These findings concur with a reported association between NMDA spikes and stimulus-specificity at the single neuron level [23] and with reports that dendritic NMDA spikes are crucial for the generation of Up-states in L5 PFC pyramidal neurons *in vitro* [20]. In support, recent studies have recorded dendritic spikes in thin dendrites *in vivo* and have correlated their appearance with a number of region-specific functions [43]–[46], indicating that dendritic events may be a brain-wide mechanism for neuronal functions.

We also claim that the minimum network size required for persistent activity induction is inversely proportional to the synaptic drive of each excitatory neuron: if synaptic input is sufficient to induce NMDA spikes, the network can be reduced down to 2 cells (albeit under unrealistic conditions for synaptic connections). A number of modeling and experimental studies have focused on the effect of network size/synaptic strength/neuronal clustering in the emergence of various physiological phenotypes [17], [47]–[49]. In fact, persistent activity was generated in very small networks but only under conditions that are far from the physiological ones [13]. This is the first study where a heavily constrained microcircuit model is used to infer a link between network size and dendritic nonlinearities with respect to persistent firing.

Finally, we find that relaxation of connectivity and synaptic delay constraints eliminates the gating effect of NMDA spikes, albeit at a cost of much larger networks. Large scale networks were classically assumed necessary for persistent activity induction [15]. Our study suggests that different mechanisms underlie persistent firing in small vs. large scale networks, at least in the PFC: NMDA-dependent dendritic spikes underlie persistent firing in small, biophysically constrained, microcircuits via the generation of long lasting somatic depolarizations; in large scale networks, these plateau potentials are replaced by massive, asynchronous inputs that are sufficient to maintain activity. Our predictions concur with the finding that intracellular application of the NMDA-channel specific blocker MK-801 in monkeys performing a working memory task abolishes persistent activity in the PFC [10], with *in vitro* studies in the visual cortex where NMDA blockade does not eliminate Up and Down states [50], as well as with *in silico* studies in large-scale networks [15] which support persistent firing without NMDA receptors, under the assumption of asynchronous spiking activity.

Our findings are particularly important from an optimization/energy conservation point of view as they suggest that active dendrites enable small microcircuits to express memory related processes such as persistent activity without requiring the recruitment of large neuronal networks and the associated energy costs.

### Mechanisms underlying persistent activity termination: New roles for old players

We found that inhibition, which was previously suggested to terminate persistent firing, mainly during Up states [22], could terminate persistent activity with a probability below 0.5. This termination could be significantly reduced by ongoing background synaptic activity as well as by the activation of the afterdepolarization mechanism (dADP) mediated by the calcium-activated non-selective cation (CAN) current. This finding points to a new functional role for the dADP, which has thus far been suggested to underlie the emergence of persistent activity [12], [23] rather than its maintenance. We claim that the dADP, regulated by neuromodulators, may play a key role in preventing interfering signals from distracting the animals and thus improving working memory performance. An experimentally testable prediction made by our model based on these findings is that termination would be more easily achieved in the absence of dADP. In addition, we predict that *in-vivo* ongoing background activity contributes to the stability of the persistent state, possibly by providing wide-spread excitation during the delay period.

### Predictive features of upcoming state transitions

Finally, we show that network activity (number of spikes) and slow synaptic mechanisms (NMDA and GABA_B_ currents), contain predictive information regarding the ability of a given stimulus to turn ON or OFF persistent firing in the microcircuit model. Interestingly, an upcoming ON state can be predicted by the microcircuit spiking activity, several milliseconds before the transition occurs. More importantly, a switch from the ON to the OFF state caused by a second inhibitory input can be predicted by the microcircuit response properties (total number of spikes) during the inducing stimulus, which is presented seconds before the termination takes place. This ability to predict ON and OFF states is in agreement with previous modeling (albeit with a different feature) work [23] and conforms with experimental work [51] showing that single neurons can encode state transitions, and PFC neurons in particular, can categorize signals *in vivo* at the onset of stimulus presentation [52]. This information is readily available to downstream regions [53], [54], presumably contributing to the preparation of a specific movement.

The predictive roles of GABA_B_ and NMDA are in accordance with recent findings that slow synaptic currents mediate persistent activity [55] and stimulus-outcome discrimination [56], respectively. The finding that both iNMDA and iGABA_B_ code for state transitions, presumably by shaping the somatic plateau potential, indicates that the balance of slow excitation/inhibition is crucial for the stability of the persistent state, as proposed by [55]. In support of this argument, we found that stable persistent activity trials were characterized by both increased NMDA and GABA_B_ currents, effectively stabilizing the microcircuit activity. This is consistent with *in vivo* experiments in the PFC where Up states are generated through a temporal enhancement of fast excitation, whereas balanced synaptic events promote their stability [57].

### Conclusions

Overall, this study zooms out from dendrites to cell assemblies and suggests a tight interaction between dendritic non-linearities and network properties (size/connectivity) that may facilitate the short-memory function of the PFC. In addition, it makes a number of novel, experimentally testable predictions regarding the role of dADP in the stability of persistent activity that may guide future studies and shed new light on memory-related processes.

## Materials and Methods

The source code of the PFC microcircuit model is available upon request to the corresponding author at poirazi@imbb.forth.gr. The model is also available via the ModelDB database (http://senselab.med.yale.edu/modeldb/ShowModel.asp?model=155057).

### Biophysical models

#### Pyramidal and interneuron models

Pyramidal and interneuron models were based on [9], implemented using the NEURON simulation environment [58]. The pyramidal model cells had a simplified morphology consisting of a soma, an axon, a basal dendrite, a proximal apical dendrite, and a distal apical dendrite. The dimensions of the somatic, axonic, and dendritic compartments are listed in Table S1. The following active mechanisms were present in the soma, proximal and distal apical dendritic tree: (a) Hodgkin–Huxley-type transient (I_Naf_), (b) persistent (I_Nap_) Na^+^ currents, (c) voltage-dependent K^+^ currents (I_Kdr_; I_A_; I_D_), (d) a fast Ca^2+^ and voltage-dependent K^+^ current (I_fAHP_), (e) a slow Ca^2+^-dependent K^+^ current (I_sAHP_), (f) a hyperpolarization-activated non-specific cation current (I_h_) and (f) four types of Ca^2+^- and voltage-dependent calcium currents (I_caN_; I_caR_; I_caL_, I_caT_). The basal dendrite included transient and persistent sodium current (I_Naf_, I_Nap_), a delayed K^+^ rectifier current (I_Kdr_), an A-type K^+^ current (I_A_), a D-type K^+^ current (I_D_), an N-type Ca^2+^ current (I_caN_) and an h current (I_h_). The axon included a transient sodium current (I_Naf_), and a delayed rectifier K^+^ current (I_Kdr_). Only in specific cases noted, the calcium-activated non-selective cation (CAN) current [23] that generates the delayed after depolarization (dADP) was activated. The L5 PFC pyramidal neuron model was validated against experimental data regarding its passive and active properties [9]. Also the kinetics and amplitude of the CAN current was fit to experimental data [9]. The passive parameters and ionic properties of the model neuron can be found in Tables S2 and S3.

The inhibitory interneuron model (fast spiking, FS) consisted of a soma and an axon. The somatic compartment included a Na^+^ current (I_Naf_) and two types of K^+^ currents (I_Kdr_; I_D_). The axon included a Na^+^ current (I_Naf_) and a delayed rectifier K^+^ currents (I_kdr_). Passive and active properties were also validated as in [9]. The dimensions of the somatic and axonic compartments are presented in Table S1. Passive and active ionic properties of the interneuron model are listed in Tables S2 and S4, respectively.

#### Microcircuit model

The microcircuit model (depicted in Figure 1A) consisted of seven pyramidal neurons and two interneurons, unless otherwise mentioned. The kinetics and amplitude of synaptic currents to both pyramidal and interneuron cells were validated under voltage clamp conditions as in [9]. Especially for the NMDA and AMPA current of pyramidal neurons, they were validated to fit the experimentally reported amplitude and kinetics between two L5 PFC pyramidal neurons (Figure S1A). We also simulated different iNMDA-to-iAMPA ratios, by enhancing the NMDA current while keeping the same AMPA current (shown in Figure S1A), since NMDA current in L5 PFC pyramidal neurons has been shown to increase by dopamine release [24], [25], due to the recruitment of extrasynaptic receptors [19] or due to glia related-processes [20]. The synaptic waveform parameters and conductances of AMPA, NMDA, GABA_A_ and GABA_B_ currents are listed in Table S5. Connectivity properties including the location and number of synaptic contacts, the latencies between pairs of neurons, as well as the electrophysiological properties of their synaptic connections, were based on anatomical and electrophysiological data (see Table S6 for parameter values). The complete mathematical formalism of the model and its components can be found in [9].

#### External stimuli

Persistent activity in the microcircuit was induced by providing external synaptic stimulation (10 pulses at 20Hz, 90 excitatory synapses) to the proximal dendrites (thick black arrow in Figure 1A) of each pyramidal model neuron [59]. The same stimulus was applied to all pyramidal neurons, as suggested by [60], [61].

#### Background noise

In order to simulate as closely as possible the noise fluctuations in the membrane potential of both pyramidal cells and interneurons that are seen *in vitro*, an artificial current with Poisson characteristics was injected in all neurons in the network.

In addition to the stochastic closing and opening of membrane ion channels, pyramidal neurons and interneurons constantly receive synaptic barrages. Few studies have investigated the properties of background synaptic input in awake, non-anesthetized animals [26], [27]. In a subset of experiments, and in order to simulate the background activity that has been reported *in vivo* during quiet wakefulness, neurons of the microcircuit (both pyramidal and interneurons) were synaptically driven by a Poisson process. In particular, we randomly distributed 120 excitatory (AMPA/NMDA) synapses at the basal, proximal apical and distal apical dendrites of all pyramidal neurons, as well as 40 synapses to the interneurons. For each trial, a single Poisson train was generated with mean value 6Hz. For each event of the Poisson train, synapses were independently activated with temporal jitter that ranged from −50 ms to +50 ms. This resulted in correlated background activity of both pyramidal neurons and interneurons, as has also been reported in [26]. On average the membrane potential of pyramidal neurons embedded in the microcircuit was at −62.9 mV and showed slow fluctuations, ranging from −73.8 mV to −43.4 mV and spontaneous spiking activity at 0.12Hz [62]. Fast spiking interneurons embedded in the microcircuit fired in the presence of background input at 12Hz [26]. Indicative traces of two pyramidal neurons from the microcircuit, in the absence of any stimulus and under iNMDA-to-iAMPA ratio 1.1 is shown in Figure S1B.

### Prediction analysis of induction and termination of persistent activity

A linear Support Vector Machine (SVM) classifier was used to examine whether certain features of the microcircuit responses (network activity features, single cell response properties and synaptic currents) could predict state transitions.

#### Features

The input features used to train and test the linear SVM classifier were: (a) single neuron inter-spike intervals (ISIs) and number of spikes during stimulus presentation, (b) the number of spikes from all the pyramidal neurons during stimulus presentation, (c) synaptic currents (iAMPA, iNMDA, iGABA_A_, iGABA_B_) measured at a single neuron by summing its synaptic current traces and (d) ratio of excitatory (iAMPA + iNMDA) transmission to inhibitory transmission (iGABA_A_ + iGABA_B_). Since traces were acquired with a sampling frequency of 10 kHz (simulation time step 0.1 ms), synaptic currents were processed using the Chebyshev Type I low pass filters in MATLAB resulting in a sampling frequency of 100Hz (dt = 10 ms). The iNMDA and iGABA_B_ traces were also processed with a low-pass Butterworth filter to eliminate fast fluctuations in order to unravel the potential impact of the slow kinetics of these currents. Both filtered and unfiltered versions of NMDA and GABA**_B_** currents were used as discriminator parameters.

#### Classification

To classify each feature matrix, a random training and test set were initially selected. The training set consisted of 100 (e.g. 50 ‘persistent’ and 50 ‘transient response’) trials. The linear SVM was then used to predict the classification of the blind test set of 30 (e.g. 15 ‘persistent’ and 15 ‘transient response’) trails. This procedure was repeated 20 times by picking randomly different training and test sets. We calculated the average prediction accuracy (number of correctly predicted trials over the 20 repetitions), the sensitivity (number of correctly predicted ‘persistent’ trials over the total number of ‘persistent’ trials) and the specificity (number of correctly predicted ‘transient response’ trials over the total number of ‘transient response’ trials) and the standard error for each prediction. The performance threshold was set to 70% (for both sensitivity and specificity) on the test set.

Data analysis was performed using MATLAB's build-in routines as well as in-house source code.

## Supporting Information

Figure S1 **Validation of the pyramidal neurons.** **A.** The iNMDA-to-iAMPA ratio was calculated by evoking an action potential to a presynaptic neuron and recording the synaptic current at the postsynaptic neuron under voltage-clamp conditions (left panel). Right panel: current traces showing the response in the soma after stimulation of a pyramidal-pyramidal pair under voltage clamp conditions at −70 mV (iAMPA) and at +60 mV and blockage of AMPA receptors (iNMDA), as in [28]. Successive traces at +60 mV correspond to the different iNMDA-to-iAMPA ratios used in this study (1.1, 1.5, 1.9 and 2.3). **B.** Voltage traces of two pyramidal neurons of the microcircuit, in the presence of background synaptic activity. Spikes are truncated for better visualization of membrane fluctuations. Note the correlated membrane potential of the two pyramidal neurons, as suggested by [26].
(TIF)

Figure S2 **iNMDA-to-iAMPA ratio supports non-linear somatic responses.** **A.** Somatic EPSP amplitude in response to two pulses at 50Hz for the 4 different ratios (red: 2.3/green: 1.9/brown: 1.5/black: 1.1) while increasing the number of activated synapses. **B.** Somatic EPSP half-width for the same conditions as in A. **C.** Somatic EPSP amplitude for the iNMDA-to-iAMPA ratio 2.3 while increasing the number of activated synapses, in response to two pulses at 50Hz (paired-red) or in response to a single pulse (single-black). **D.** Somatic EPSP half-width for the same conditions as in C. **E.** Experimental somatic recordings from L5 PFC pyramidal neurons, after stimulation of the basal dendrites with a single (black) or two pulses at 50Hz (red). Adapted with permission from [19].
(TIF)

Figure S3 **Synaptic currents as predictive features.** **A.** Indicative trace of the iGABA_B_ during the presentation of the inducing stimulus as well as the trace obtained after filtering (smooth line). **B.** Indicative trace of the iNMDA during the presentation of the inducing stimulus as well as the trace obtained after filtering (smooth line). **C.** Box plot showing the summed iGABA_B_ response during the 500 ms of stimulus presentation, when persistent activity was not induced (transient response) and when persistent activity emerged (persistent). **D.** Box plot showing the summed iGABA_B_ response during the 500 ms of stimulus presentation, when persistent activity was maintained (stable persistent) and when persistent activity was terminated (terminated persistent). **E.** Box plot showing the summed iNMDA response during the 500 ms of stimulus presentation, when persistent activity was not induced (transient response) and when persistent activity emerged (persistent). **F.** Box plot showing the summed iNMDA response during the 500 ms of stimulus presentation, when persistent activity was maintained (stable persistent) and when persistent activity was terminated (terminated persistent).
(TIF)

Figure S4 **Somatic depolarization plateau and spiking activity support excitable states.** Box plot of the mean depolarizing potential during the last 100 ms of the stimulus presentation calculated over all pyramidal neurons. **A.** The magnitude of the depolarizing potential for trials in which persistent activity was not induced (transient response) and for trials where persistent activity emerged (persistent). **B.** Same for trials whereby persistent activity was terminated and trials in which persistent activity was maintained (stable persistent). **C.** Box plot showing the total number of spikes from all 7 pyramidal neurons during the 500 ms of stimulus presentation, when persistent activity was not induced (transient response) and when persistent activity emerged (persistent). **C.** Box plot showing the total number of spikes from all 7 pyramidal neurons during the 500 ms of stimulus presentation, when persistent activity was maintained (stable persistent) and when persistent activity was terminated.
(TIF)

Table S1 **Structure of model cells.**
(DOCX)

Table S2 **Passive properties of pyramidal cells and inhibitory interneurons in the microcircuit.**
(DOCX)

Table S3 **Active ionic properties of pyramidal neurons.**
(DOCX)

Table S4 **Active ionic properties of inhibitory interneurons.**
(DOCX)

Table S5 **Synaptic parameters.**
(DOCX)

Table S6 **Summary of synaptic connections in the microcircuit.**
(DOCX)
