# Supplementary figures and images for: Dendritic Nonlinearities Reduce Network Size Requirements and Mediate ON and OFF States of Persistent Activity in a PFC Microcircuit Model
Source: PLoS Comput Biol. 2014 Jul 31;10(7):e1003764. doi: 10.1371/journal.pcbi.1003764 (PMC4117433; doi:10.1371/journal.pcbi.1003764)

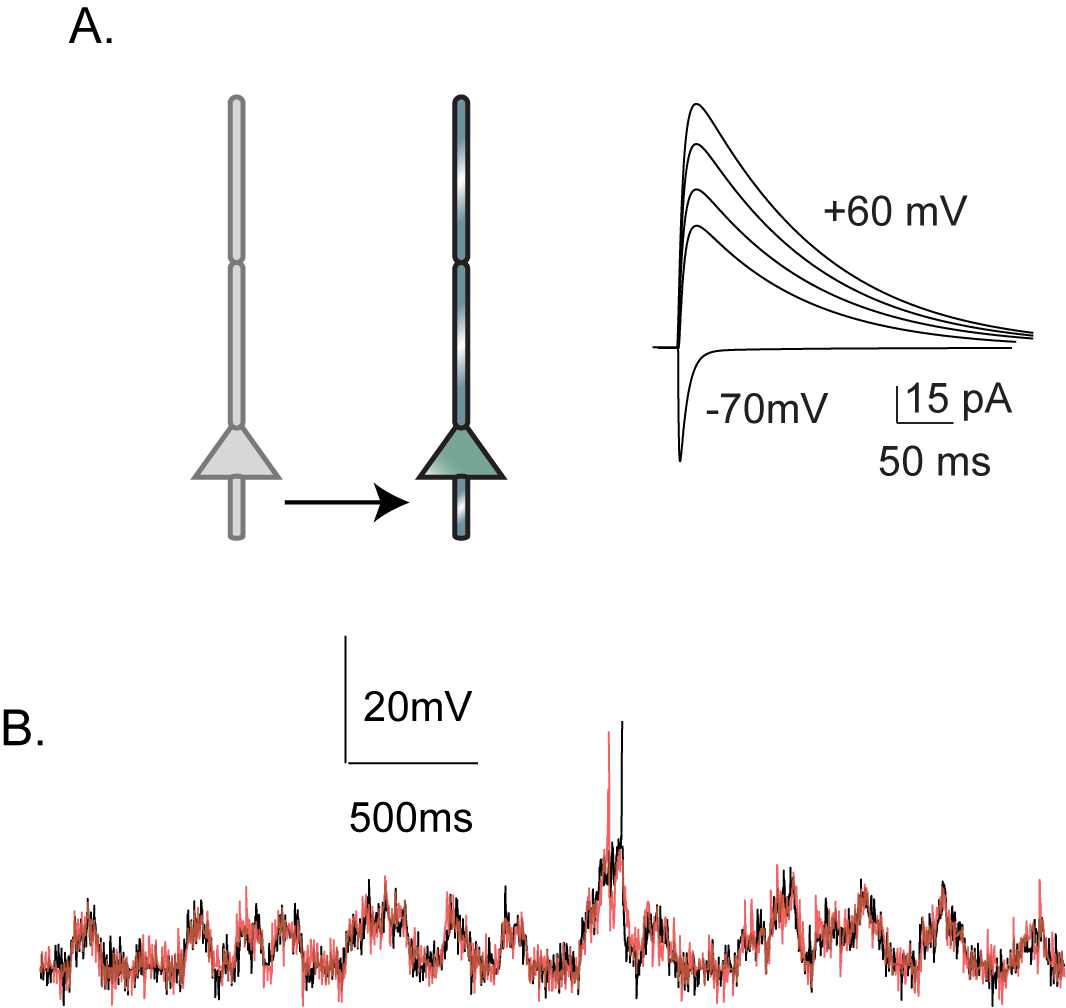

Supplement: Figure S1 — Validation of the pyramidal neurons. A. The iNMDA-to-iAMPA ratio was calculated by evoking an action potential to a presynaptic neuron and recording the synaptic current at the postsynaptic neuron under voltage-clamp conditions (left panel). Right panel: current traces showing the response in the soma after stimulation of a pyramidal-pyramidal pair under voltage clamp conditions at −70 mV (iAMPA) and at +60 mV and blockage of AMPA receptors (iNMDA), as in [28]. Successive traces at +60 mV correspond to the different iNMDA-to-iAMPA ratios used in this study (1.1, 1.5, 1.9 and 2.3). B. Voltage traces of two pyramidal neurons of the microcircuit, in the presence of background synaptic activity. Spikes are truncated for better visualization of membrane fluctuations. Note the correlated membrane potential of the two pyramidal neurons, as suggested by [26]. (TIF) [file pcbi.1003764.s001.tif]

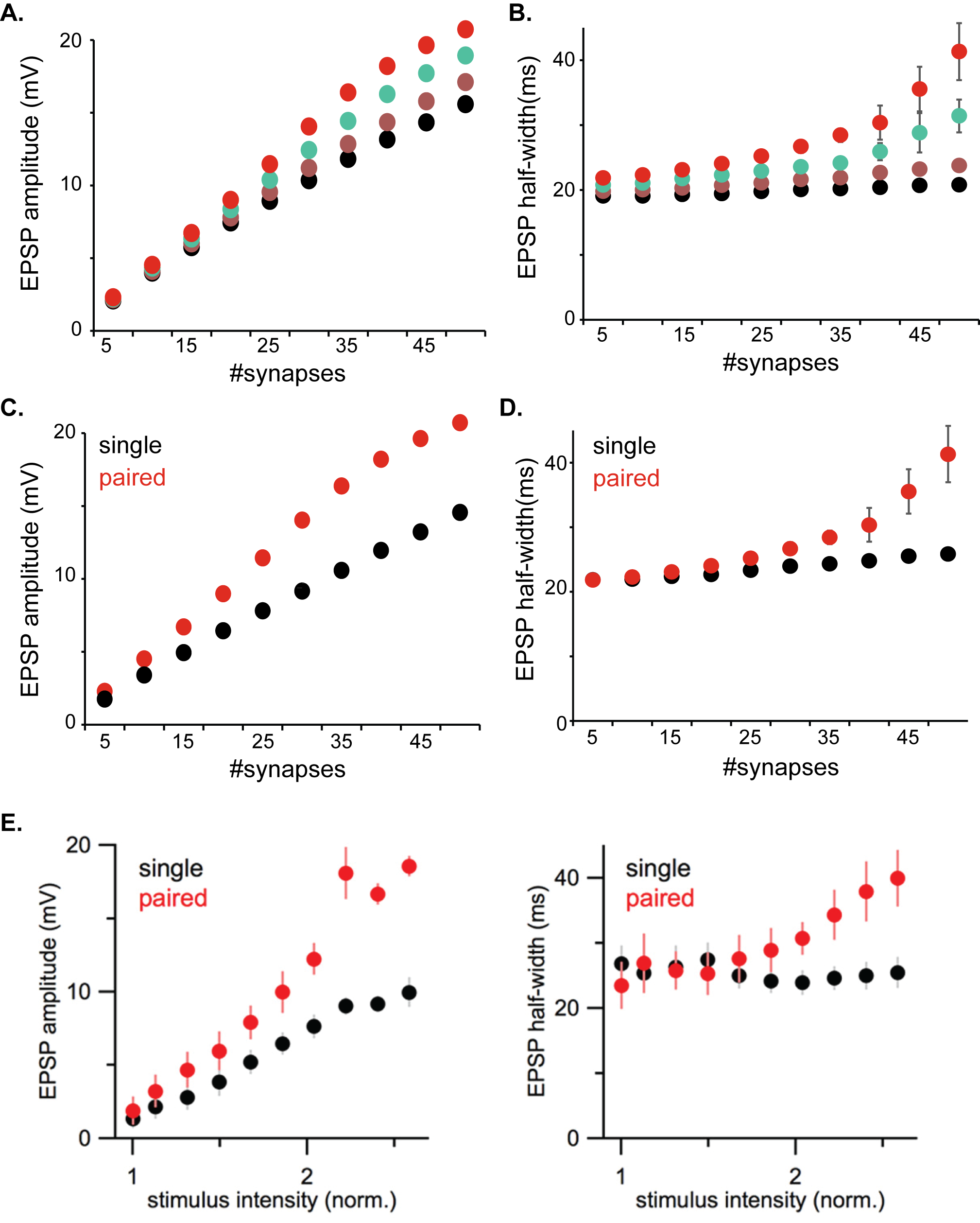

Supplement: Figure S2 — iNMDA-to-iAMPA ratio supports non-linear somatic responses. A. Somatic EPSP amplitude in response to two pulses at 50Hz for the 4 different ratios (red: 2.3/green: 1.9/brown: 1.5/black: 1.1) while increasing the number of activated synapses. B. Somatic EPSP half-width for the same conditions as in A. C. Somatic EPSP amplitude for the iNMDA-to-iAMPA ratio 2.3 while increasing the number of activated synapses, in response to two pulses at 50Hz (paired-red) or in response to a single pulse (single-black). D. Somatic EPSP half-width for the same conditions as in C. E. Experimental somatic recordings from L5 PFC pyramidal neurons, after stimulation of the basal dendrites with a single (black) or two pulses at 50Hz (red). Adapted with permission from [19]. (TIF) [file pcbi.1003764.s002.tif]

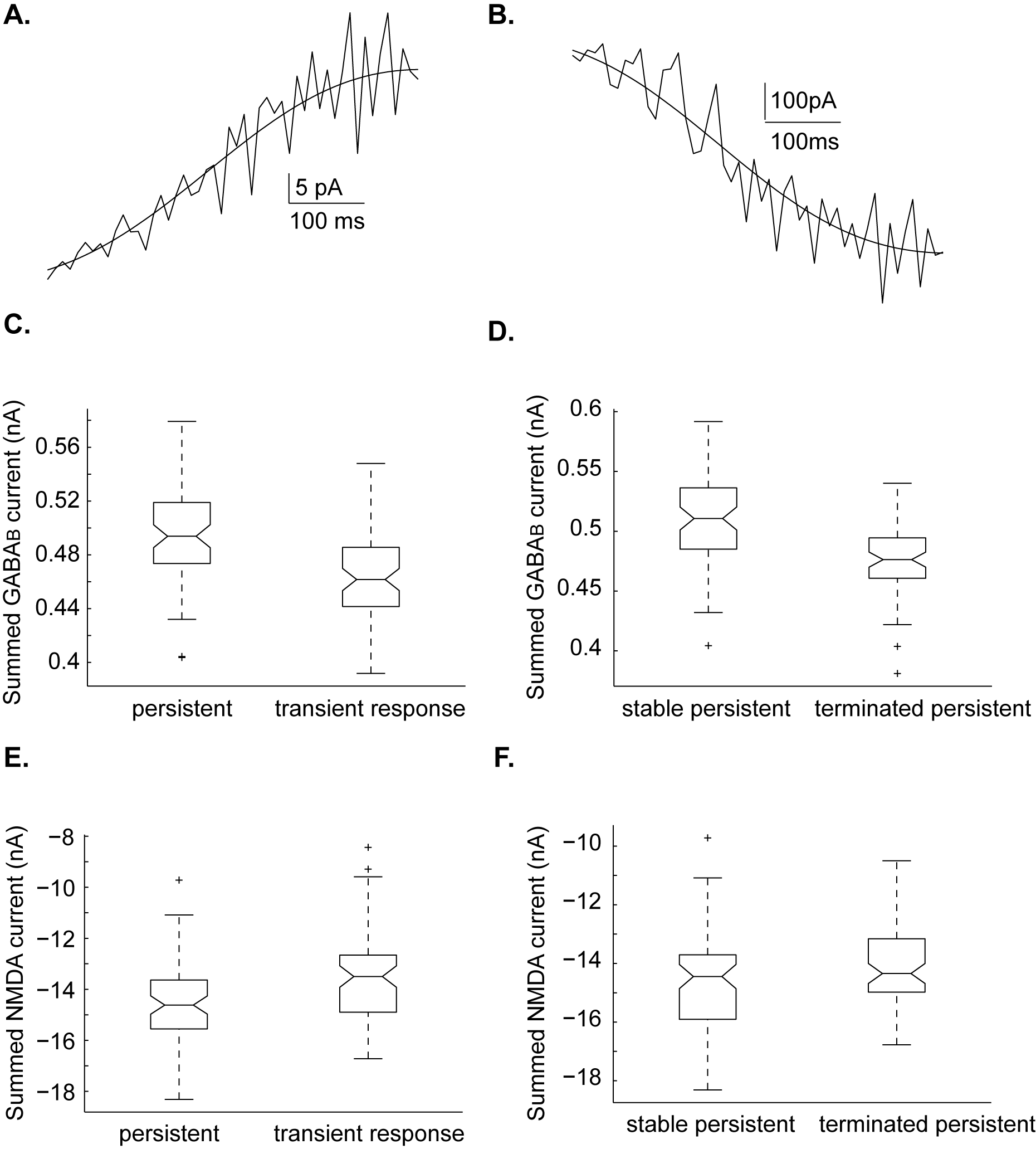

Supplement: Figure S3 — Synaptic currents as predictive features. A. Indicative trace of the iGABAB during the presentation of the inducing stimulus as well as the trace obtained after filtering (smooth line). B. Indicative trace of the iNMDA during the presentation of the inducing stimulus as well as the trace obtained after filtering (smooth line). C. Box plot showing the summed iGABAB response during the 500 ms of stimulus presentation, when persistent activity was not induced (transient response) and when persistent activity emerged (persistent). D. Box plot showing the summed iGABAB response during the 500 ms of stimulus presentation, when persistent activity was maintained (stable persistent) and when persistent activity was terminated (terminated persistent). E. Box plot showing the summed iNMDA response during the 500 ms of stimulus presentation, when persistent activity was not induced (transient response) and when persistent activity emerged (persistent). F. Box plot showing the summed iNMDA response during the 500 ms of stimulus presentation, when persistent activity was maintained (stable persistent) and when persistent activity was terminated (terminated persistent). (TIF) [file pcbi.1003764.s003.tif]

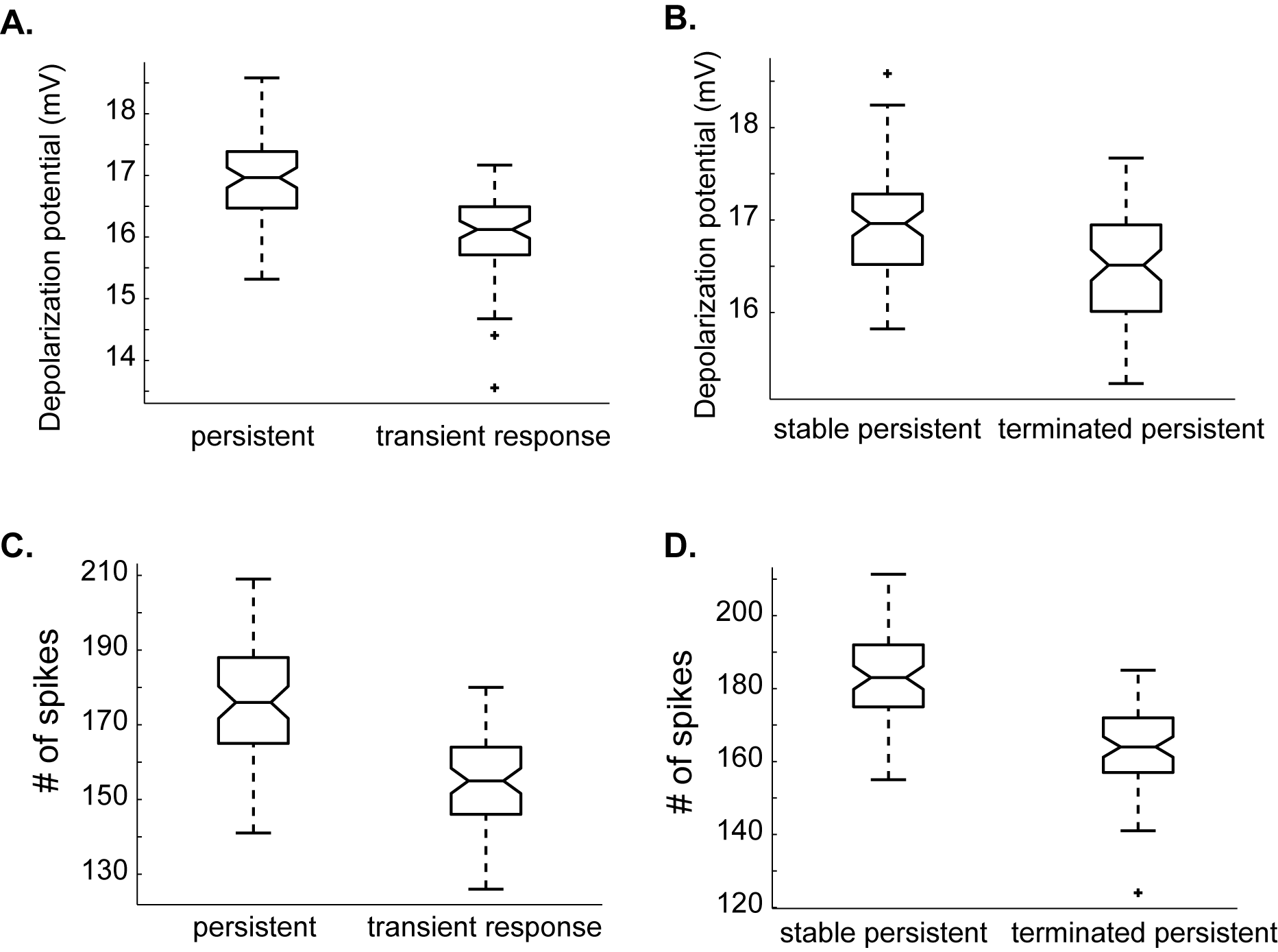

Supplement: Figure S4 — Somatic depolarization plateau and spiking activity support excitable states. Box plot of the mean depolarizing potential during the last 100 ms of the stimulus presentation calculated over all pyramidal neurons. A. The magnitude of the depolarizing potential for trials in which persistent activity was not induced (transient response) and for trials where persistent activity emerged (persistent). B. Same for trials whereby persistent activity was terminated and trials in which persistent activity was maintained (stable persistent). C. Box plot showing the total number of spikes from all 7 pyramidal neurons during the 500 ms of stimulus presentation, when persistent activity was not induced (transient response) and when persistent activity emerged (persistent). C. Box plot showing the total number of spikes from all 7 pyramidal neurons during the 500 ms of stimulus presentation, when persistent activity was maintained (stable persistent) and when persistent activity was terminated. (TIF) [file pcbi.1003764.s004.tif]
