# Supplementary material for: Dendritic Nonlinearities Reduce Network Size Requirements and Mediate ON and OFF States of Persistent Activity in a PFC Microcircuit Model
Source: PLoS Comput Biol. 2014 Jul 31;10(7):e1003764. doi: 10.1371/journal.pcbi.1003764 (PMC4117433; doi:10.1371/journal.pcbi.1003764)
Supplement: Table S1 — Structure of model cells. (DOCX) [file pcbi.1003764.s005.docx]

**Table S1.** Structure of model cells

|  | **Length (μm)** | **Diameter (μm)** |
| --- | --- | --- |
| **Pyramidal cell** | | |
| Soma | 75 | 10.14 |
| Basal dendrite | 150 | 1 |
| Proximal apical dendrite | 400 | 3.4 |
| Distal apical dendrite | 400 | 2.6 |
| Axon | 113.22 | 1.1 |
| **Inhibitory interneuron** | | |
| Soma | 53 | 42 |
| Axon | 113.22 | 0.7 |
