# Supplementary material for: Dendritic Nonlinearities Reduce Network Size Requirements and Mediate ON and OFF States of Persistent Activity in a PFC Microcircuit Model
Source: PLoS Comput Biol. 2014 Jul 31;10(7):e1003764. doi: 10.1371/journal.pcbi.1003764 (PMC4117433; doi:10.1371/journal.pcbi.1003764)
Supplement: Table S2 — Passive properties of pyramidal cells and inhibitory interneurons in the microcircuit. (DOCX) [file pcbi.1003764.s006.docx]

**Table S2.** Passive properties of pyramidal cells and inhibitory interneurons in the microcircuit.

|  | **C_M_, μF/cm^2^** | **R_A_, ohm/cm** | **R_M_, kΩ cm^2^** |
| --- | --- | --- | --- |
| **Pyramidal cell** | | | |
| Soma | 1.2 | 100 | 16 |
| Basal dendrite | 2 | 100 | 5.9 |
| Proximal apical dendrite | 1.2 | 150 | 5.9 |
| Distal apical dendrite | 1.2 | 150 | 5.9 |
| Axon | 1.2 | 150 | 12 |
| **Inhibitory interneuron** | | | |
| Soma | 1.2 | 150 | 15 |
| Axon | 1.2 | 150 | 15 |
