# Supplementary material for: Dendritic Nonlinearities Reduce Network Size Requirements and Mediate ON and OFF States of Persistent Activity in a PFC Microcircuit Model
Source: PLoS Comput Biol. 2014 Jul 31;10(7):e1003764. doi: 10.1371/journal.pcbi.1003764 (PMC4117433; doi:10.1371/journal.pcbi.1003764)
Supplement: Table S3 — Active ionic properties of pyramidal neurons. (DOCX) [file pcbi.1003764.s007.docx]

**Table S3.** Active ionic properties of pyramidal neurons

| **Mechanisms** | **Soma** | **Axon** | **Basal**  **Dendrites** | **Proximal Apical dendrites** | **Distal Apical dendrites** |
| --- | --- | --- | --- | --- | --- |
| Sodium conductance, S/cm^2^ | 0.1809 | 0.18 | 0.0018 | 0.005 | 0.0036 |
| Delayed rectifier K^+^, S/cm^2^ | 0.0216 | 0.0054 | 0.0054 | 2.16e-5 | 5.4e-6 |
| Persistent sodium, S/cm^2^ | 0.18e-5 | 0 | 1.8e-5 | 5.4e-5 | 1.8e-4 |
| A-type K^+^, S/cm^2^ | 7e-4 | 0 | 7e-4 | 7e-5 | 7e-5 |
| D-type K^+^, S/cm^2^ | 1.026e-3 | 0 | 6e-4 | 1.2e-3 | 1.2e-3 |
| N-type calcium, S/cm^2^ | 2e-5 | 0 | 6e-5 | 6e-5 | 1e-3 |
| T-type calcium, S/cm^2^ | 6e-6 | 0 | 0 | 6e-5 | 6e-6 |
| CaR, S/cm^2^ | 3e-5 | 0 | 0 | 9e-6 | 1.5e-3 |
| L-type calcium, S/cm^2^ | 3e-5 | 0 | 0 | 1.9e-4 | 3.6e-6 |
| sAHP, S/cm^2^ | 1.4e-1 | 0 | 0 | 2.75e-3 | 2.75e-5 |
| fAHP, S/cm^2^ | 2.2e-3 | 0 | 0 | 2.2e-5 | 2.2e-6 |
| H-current, S/cm^2^ | 7.2e-6 | 0 | 9e-6 | 1.4e-5 | 9e-5 |
| dADP current, S/cm^2^ | 0 | 0 | 0 | 0 | 0 |
| Calcium diffusion model | Yes | No | Yes | Yes | Yes |
| E_na_, mV | +55 | +55 | +55 | +55 | +55 |
| E_K_, mV | -80 | -80 | -80 | -80 | -80 |
| E_ca_, mV | +140 | - | +140 | +140 | +140 |
| E_h_, mV | -10 | - | -10 | -10 | -10 |
| E_L_, mV | -65 | -65 | -65 | -65 | -65 |
