# Supplementary material for: Dendritic Nonlinearities Reduce Network Size Requirements and Mediate ON and OFF States of Persistent Activity in a PFC Microcircuit Model
Source: PLoS Comput Biol. 2014 Jul 31;10(7):e1003764. doi: 10.1371/journal.pcbi.1003764 (PMC4117433; doi:10.1371/journal.pcbi.1003764)
Supplement: Table S4 — Active ionic properties of inhibitory interneurons. (DOCX) [file pcbi.1003764.s008.docx]

**Table S4.** Active ionic properties of inhibitory interneurons

| **Mechanisms** | **Soma** | **Axon** |
| --- | --- | --- |
| Sodium conductance, S/cm^2^ | 0.225 | 0.54 |
| Delayed rectifier, S/cm^2^ | 0.018 | 0.018 |
| D-type K^+^, S/cm^2^ | 7.25e-5 | 0 |
| E_na_, mV | +55 | +55 |
| E_K_, mV | -80 | -80 |
| E_L_, mV | -70 | -70 |
