# Supplementary material for: Dendritic Nonlinearities Reduce Network Size Requirements and Mediate ON and OFF States of Persistent Activity in a PFC Microcircuit Model
Source: PLoS Comput Biol. 2014 Jul 31;10(7):e1003764. doi: 10.1371/journal.pcbi.1003764 (PMC4117433; doi:10.1371/journal.pcbi.1003764)
Supplement: Table S5 — Synaptic parameters. (DOCX) [file pcbi.1003764.s009.docx]

**Table S5.** Synaptic parameters

|  | **AMPA** | **NMDA** | **GABA_A_** | **GABA_B_** |
| --- | --- | --- | --- | --- |
| **Pyramidal cell** | | | | |
| Conductance, nS | 0.19 (basal)  0.24 (apical) | 0.25 (basal)  0.22 (apical) | 6.9e-4 | 1.05e-4 |
| Reversal potential, mV | 0 | 0 | -80 | -80 |
| Rise time, ms | 0.6^*^ | 4.3 | 1.5 | 9.8 |
| Fall time, ms | 4.3^*^ | 93 | 14 | 72 |
| **Inhibitory interneuron** | | | | |
| Conductance, S/cm^2^ | 7.5e-4 | 3.2e-4 | 5.1e-4 | - |
| Reversal potential, mV | 0 | 0 | -80 | - |
| Rise time, ms | 0.3^*^ | 0.5^*^ | 3 | - |
| Fall time, ms | 5.5^*^ | 66.3^*^ | 24 | - |

^*^Data adapted from voltage-clamp recordings
