# Supplementary material for: Dendritic Nonlinearities Reduce Network Size Requirements and Mediate ON and OFF States of Persistent Activity in a PFC Microcircuit Model
Source: PLoS Comput Biol. 2014 Jul 31;10(7):e1003764. doi: 10.1371/journal.pcbi.1003764 (PMC4117433; doi:10.1371/journal.pcbi.1003764)
Supplement: Table S6 — Summary of synaptic connections in the microcircuit. (DOCX) [file pcbi.1003764.s010.docx]

**Table S6.** Summary of synaptic connections in the microcircuit.

| **Type of connection** | **Location** | **# of synapses** | **Reference** |
| --- | --- | --- | --- |
| Thalamocortical (incoming) | Proximal dendrite | 50 | (Kuroda M *et al.*, 1998) |
| Pyramidal recurrent | Basal dendrite | 5 | (Markram et al., 1997) |
| Pyramidal-to-interneuron | Soma | 2 | (Buhl *et al.*, 1997) |
| Autapses in pyramidal neurons | Basal dendrite | 1 | (Lübke et al., 1996; Tamás et al., 1997b) |
| Interneuron-to-pyramidal | Soma | 4 | (Tamás et al., 1997a) |
| Autapses in the interneuron | Soma | 12 synaptic contacts producing ~350pA | (Tamás et al., 1997b; Bacci et al., 2003) |
